# Validation of portable tablets for transplant pathology diagnosis according to the College of American Pathologists Guidelines

**DOI:** 10.1016/j.acpath.2022.100047

**Published:** 2022-07-31

**Authors:** Stefano Marletta, Liron Pantanowitz, Deborah Malvi, Luca Novelli, Claudia Mescoli, Massimo Cardillo, Antonietta D'Errico, Ilaria Girolami, Albino Eccher

**Affiliations:** a Department of Pathology and Diagnostics and Public Health, Section of Pathology, University Hospital of Verona, Verona, Italy; b Department of Pathology & Clinical Labs, University of Michigan, Ann Arbor, MI, USA; c Department of Specialized, Experimental and Diagnostic Medicine, Pathology Unit, IRCCS, Azienda Ospedaliero-Universitaria di Bologna, Bologna, Italy; d Institute of Histopathology and Molecular Diagnosis, Careggi University Hospital, Florence, Italy; e Surgical Pathology and Cytopathology Unit, Department of Medicine, University and Hospital Trust of Padua, Padua, Italy; f National Transplant Center, Rome, Italy; g Division of Pathology, Central Hospital, Bolzano, Italy; h Department of Pathology and Diagnostics, University and Hospital Trust of Verona, Verona, Italy

**Keywords:** Digital pathology, Transplantation pathology, Validation, Tablets

## Abstract

Despite increased use of digital pathology, its application in the transplantation setting remains limited. One of the restraints is related to concerns that this technology is inadequate for supporting diagnostic work. In this study, we sought to establish non inferiority of whole slide imaging (WSI) to light microscopy (LM) for intraoperative transplantation diagnosis using inexpensive portable devices. A validation study was conducted according to updated guidelines from the College of American Pathologists (CAP) utilizing 80 intraoperative transplantation cases. Two pathologists reviewed glass slides with LM and digital slides on two different tablets after a washout period of 4 weeks. Diagnostic concordance and intra-observer agreement were recorded. A total of 45 (56%) cases were suitable for rendering transplant diagnoses and 35 (44%) for assessing cancer risk. Intra-observer agreement was 95.1% for organ suitability and 100% for cancer risk. There were no major discordances that could affect patient transplant management. Digital evaluation of intraoperative transplant specimens using tablets to view whole slide images was non-inferior to LM for primary diagnosis. This suggests that after validating WSI these digital tools can be safely used for remote intraoperative transplantation diagnostic work.

## Introduction

Whole slide imaging (WSI) refers to scanning (digitization) of entire glass slides (GSL) to acquire and view their digitized version (digital slides) on a computer monitor, thus virtually simulating light microscopy (LM).^1^ Alongside letting physicians navigate and efficiently analyze virtual slides, archiving WSIs permits sharing of digital files via telepathology for both supporting primary diagnosis at remote locations and for rapidly getting a second opinion on challenging cases via teleconsultation. A number of regulatory bodies, both in Europe and North America, have already approved the use of WSI for primary diagnostic purposes.^2^

Various guidelines have been established that help pathology laboratories validate WSI for diagnostic use in clinical practice. The College of American Pathologists (CAP) first developed such guidelines in 2013^3^ and recently updated them in 2021.^4^ They include three strong recommendations (including a validation set of at least 60 cases, employing a washout interval longer than 2 weeks, and achieving an overall WSI-GSL concordance ≥95%), and nine Good Practice Statements (GPS). Validation is defined as a process that demonstrates WSI will perform as expected for its intended use. Indeed, many laboratories around the world have internally validated their WSI systems to diagnose routine surgical pathology cases,^5^ frozen section intraoperative diagnoses,^6^^,^^7^ and interpretation of transplantation cases.^8^^,^^9^ As the majority of practicing pathologists have limited expertise with transplantation pathology, this field initially faced difficulties embracing WSI technology.^10^ Nevertheless, the application of digital pathology in this setting offers great potential with pre-transplant specimen procurement and during the donor evaluation phase of transplantation. The management of donors with newly discovered cancer requires a timely pathology diagnosis based on guidelines that stratify the risk of cancer transmission.^11^, ^12^, ^13^, ^14^, ^15^ This demand can be addressed by leveraging telepathology to gain access to pathology transplantation specialists.^16^^,^^17^

Several challenges such as the expensive cost of WSI equipment have been a major limiting factor for the widespread adoption of digital pathology. To date, there have been only a few studies documenting the use of affordable devices such as tablets and smartphones instead of expensive medical grade monitors in pathology.^18^^,^^19^ Employing such low-cost tools in the transplantation setting is still anecdotal.^20^ Thus, the aim of this study was to test the performance of two inexpensive portable tablets by validating their diagnostic use in transplant pathology according to the CAP guidelines.

## Materials and methods

### Case enrollment

Following approval from the institutional ethics committee, consecutive hematoxylin and eosin (H&E) stained GSL used for routine diagnostic use in transplantation pathology, accessioned between June 2021 and December 2021, were retrieved from the archives of the Pathology Laboratory of Bologna. Eighty consecutive cases were collected. After enrollment in the validation set, these GSL were de-identified and assigned a unique study identification number.

### Ethics approval and consent

Patients were not required to give informed consent to the study because the analysis used anonymous clinical data that were obtained after each patient agreed to treatment by written consent.

### Imaging hardware and software

**Fig. 1:**
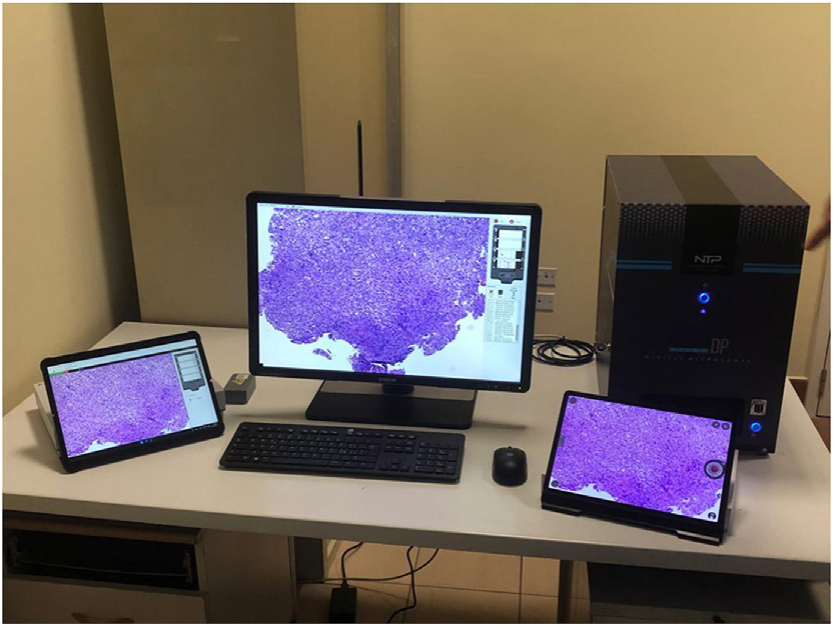
Fixed digital workstation (scanner & monitor) and the 2 portable monitors for remote viewing.

The selected slides were then used to acquire whole slide digital images using a NTP NED.Micro.DP® microscope-based scanner at 40× magnification with a resolution of 0.25 μm/pixel set on automated mode for tissue detection, as per the manufacturer's instructions. This instrument contains an in-built computer system with 32 GB internal storage and preinstalled image viewing software, image server, and web server. The instrument was operated through a laptop device with an Intel® Atom™ x7-E3950 Quad Core @1.6 GHz (Burst 2.0 GHz) 8 GB RAM memory processor and a 64-bit operating system (Microsoft® Windows 10 Enterprise). Acquired WSIs were simultaneously available for visualization both on a computer display and on two different models of tablets. As for the former, an Eonis® (MDRC-2224 BL) LCD flat panel monitor with 24.1″ screen size was employed, characterized by a color depth of 10-bit, 1920 × 1200 display resolution, brightness power of 300 cd/m^2^ and a contrast ratio of at least 1000:1. With regard to the two portable devices, the first was a Microsoft Surface Pro X ® tablet with a 13″ touchscreen brilliant PixelSense™ display and a 2880 × 1920 monitor resolution operated using Microsoft SQ 2 ® software, and 16 GB RAM memory operating on a 64-bit Windows 10 Home on ARM system. The second portable tablet was a Samsung Galaxy Tab S7 FE 5G® with a 12.4″ LTPS TFT screen and 2560 × 1600 display resolution using Qualcomm Snapdragon 865 Plus software and an Octa Core (3.09 GHz + 2.4 GHz + 1.8 GHz) processor; this latter tablet had 4 GB RAM memory operating with an Android 10 system (Fig. 1). Technical characteristics of the devices are summarized inSupplementary Table S1.

### Definition of major and minor diagnostic discordances

The definition of major and minor discordance was tailored according to the specific clinical setting of transplantation. There are two types of questions for pathologists to answer during the transplantation process: (i) suitability of the organ for transplant, and (ii) nature of a lesion found in the potential donor by the surgeons, and the resultant risk this has for malignancy transmission.

The suitability of organs was evaluated with semiquantitative scores for liver fibrosis (Ishak score), percentage of liver steatosis, and the Remuzzi score for kidney biopsy.^21^ In this setting, a major discordance is considered when the score rendered based upon the LM and the score derived from the WSI would have led to a change in allocation (e.g. organ discarded because of a higher score) or conversely resulted in improper allocation (e.g. because of a much lower score). A minor discordance is when the score or the percentage of steatosis are different, but are in the same range and would have not changed the indication for organ suitability.

The risk of malignancy transmission was graded according to the Italian National transplant center (CNT) guidelines.^22^ This included standard risk for donors with benign lesions, negligible risk for those with some type of neoplastic lesions with a very low potential of transmission, acceptable risk for cases with significant chance of transmission but still to be transplanted for specific situations, and unacceptable risk for overtly malignant lesions with concrete risk of transmission. In this context, a major discordance is considered when the assessment based upon the WSI would have changed the risk category, thus leading to a relevant change in donor management, while a minor discordance is employed when the lesion is differently described or categorized but there is no change in the risk category.

### Diagnostic assessment: WSI versus LM

**Fig. 2:**
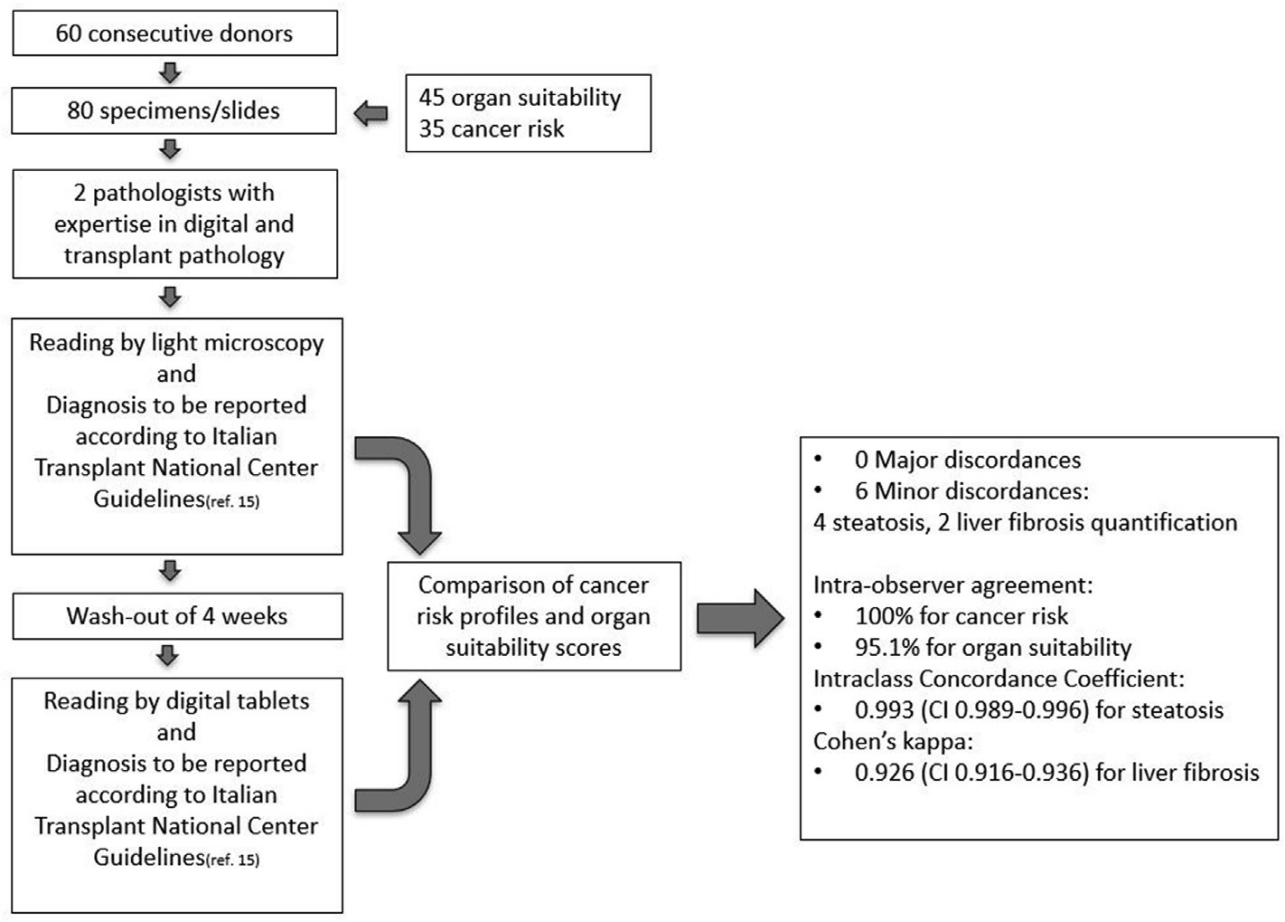
Schematic workflow of the validation study.

Study design is summarized in Fig. 2, which depicts the validation and study accrual workflow. The validation set was assessed by two pathologists experienced in digital pathology (AE, AD), which for the purposes of this study we defined as having both long-standing expertise in the routine use of digital slides, and having contributed substantively to the scientific peer-review literature of digital pathology. These two pathologists initially reviewed the original GSLs by conventional LM and then the corresponding WSIs on a digital screen of either one of the tablets, following a proper washout interval of 4 weeks. The two pathologists reviewed the cases in order to classify them according to risk profiles (cancer risk) or to provide scores (organ suitability) and to make sure that LM and WSI results were comparable. The first investigator used the Microsoft Surface Pro X ® tablet while the other one reviewed the virtual slides on the Samsung Galaxy Tab S7 FE 5G ® device. Following the validation process, and to ensure that this study was based on established subspecialty expertise in transplantation pathology, experts in heart, kidney, and liver transplant pathology (AE, AD, CM, DM, LN) provided the diagnostic interpretations for the formal accrual of study data. Pathologists were provided with relevant clinical-radiological information available at the time of the on-call transplantation consultation and were blinded to the original signed out diagnosis, which was considered the reference standard. Each diagnosis rendered by the reading pathologist on a case (whether by WSI or LM) was considered a “read” so that there were 4 “reads” per case, besides the reference (sign-out) diagnosis. Concordance rates were calculated separately for the category of suitability and risk of malignancy, while for categories of semiquantitative scores the ĸ Cohen index with a 95% CI was chosen for estimating intra-observer and inter-observer agreement between WSI scores and those made by GSL, according to the following classification: no to slight (0,00-0,20), fair (0,21-0,40), moderate (0,41-0,60), substantial (0,61-0,80), and excellent agreement (≥0.81). Disagreements between diagnoses rendered with the two different methods were classified as either minor or major discordances considering whether they could have a relevant impact on overall clinical management or not.

## Results

### Case population

There was a total of 80 slides from 60 donors (34 male and 26 female), with a median age of 75 years (range 44–89). Each donor provided 1 to 5 specimens to the case population. There were 45 (56%) cases to be assessed for suitability to transplant and 35 (44%) for determination of cancer risk. Among the suitability cases, there were 39 liver biopsies and 6 kidney biopsies, while among the oncological cases in 18 (51%) cases only the lesion of interest was sent for examination, in 16 (46%) the whole organ was sent and in 1 (3%) case only a biopsy specimen of the lesion was sent. In the overall population, the distribution of sites of specimens was as follows: gastrointestinal 49 (61%), genitourinary 12 (15%), endocrine organs 7 (9%), respiratory 5 (6%), gynecological 5 (6%), 1 lymphopoietic (1%), and 1 peritoneal (1%). The cancer risk group comprised 5 lung cases, 5 thyroid gland, 4 ovarian, 3 prostate, 3 pancreas, 2 gallbladder, 2 bladder, 2 adrenal gland, 2 small intestine, 2 liver, and 1 case each for stomach, kidney, peritoneum, uterus and lymph node.

### Concordance between LM and WSI

Concerning the suitability to transplant cases, there we no major discordances between the reading of the two pathologists with the two modalities. Only for liver biopsy cases, we found some minor discordances, which are detailed as follows: 2 out of 39 (5%) livers showed minor discordance in fibrosis assessment and 4 out of 39 (10%) showed minor discordance in steatosis quantification. The intraclass correlation coefficient (ICC) for the continuous value of steatosis was 0.993 (CI 0.989–0.996) for both readings, while the Cohen's kappa for the fibrosis score was 0.926 (CI 0.916–0.936) for both readings.

Concerning the cancer risk group of cases, there were no major discordances and the distribution of risk categories was the same for both readings: 26 (74%) standard, 4 (12%) negligible, 3 (9%) unacceptable, and 2 (6%) acceptable.

Clinicopathological features of the cases of the present study along with concordance comparison between LM and digital images are listed in Supplementary Tables S2, S3, and S4.

## Discussion

The CAP recently updated their originally released guidelines for validation of digital pathology systems for primary diagnostic use, advocating that pathology laboratories adhere to their proposed recommendations to ensure that this technology is safe for routine pathology practice. Specifically, the Good Practice Statement n. 6 of the 2021 CAP guidelines^4^ states that pathologists adequately trained to use the WSI system must be involved in the validation process. The CAP does not provide evidence-based recommendations about the type of training, or the metrics used to determine technical competency of pathologists using WSI systems. As such, adequate training is defined at the discretion of the laboratory medical director. For the purposes of this study, a very high level of digital pathology expertise was brought to this study, both for validation of the digital pathology system to be used, and for the actual performance of the prospective study.

Despite several institutions having fully embraced telepathology for getting second opinion teleconsultations in a timely manner,^23^ global widespread adoption of digital pathology remains limited.^24^ Due to the need for rapid turn-around-times and expert pathology interpretation for proper organ allocation, the transplantation setting would greatly benefit from accessible teleconsultation services, as most institutions usually lack dedicated transplantation pathologists. Not surprisingly, internationally recognized transplantation working groups have started to encourage the use of reliable digital pathology systems to support urgent second opinions and reproducible image analysis-based evaluation of harvested specimens.^25^ Hence, in the present study we validated two affordable and easy-to-use tablets for primary pre-transplantation diagnosis, following the guidelines provided by the CAP.

Compared to LM, after a washout period of 4 weeks, both digitally-experienced pathologists achieved a 100% intra-observer agreement for all of the specimens submitted for cancer risk assessment, and a 95.1% intra-observer agreement when dealing with organs sent for assessment of graft suitability. This latter value was only slightly affected by a few of minor discordances. Disagreements between WSI and LM were attributed to discordances that would not have negatively impacted the clinical management of the transplant. Of all the organs sent for suitability assessment, the few cases where discordances were exclusively recorded pertained to the estimation of liver biopsy fibrosis and steatosis. By comparison, complete agreement was reached for evaluation of kidney quality when utilizing the Karpinski-Remuzzi grading system. Only marginal discrepancies were observed with both tablets, indicative of the lack of technical issues with these devices in this study. The discrepancies noted are more likely due to challenging nature of the specimens, as frozen sections from liver biopsies may often harbor technique-related artifacts potentially hampering the evaluation of steatosis and fibrosis.^26^

The series in this study included 80 transplantation cases, which is more than the suggested amount of 60 cases recommended in the CAP validation guidelines. The CAP guidelines recommend including in the validation set cases that represent the spectrum of diagnoses likely to be encountered in routine daily practice. This is of particular concern in the transplantation field, as the current shortage of organs has increasingly led to frequent consideration of grafts from so-called “marginal donors”. These donors could either be (i) subjects with less favorable clinical history and function (so-called expanded criteria donors, ECD) or (ii) donors with a previous history of malignancy or with a neoplastic process discovered at the time of donor evaluation. About the former, several studies have demonstrated comparable clinical outcomes for accurately selected recipients,^27^^,^^28^ and nowadays intraoperative histological evaluation is usually the final exam for choosing between organs to transplant or discard.^21^ Thus, if such demanding diagnostic decisions are to be made digitally then the WSI-based validation process ought to demonstrate non-inferiority compared to conventional LM for this task.^29^^,^^30^

A notable proportion of cases from our series (56%) was indeed composed of specimens from ECD which showed only a few minor discordances between LM and WSI, achieving the goal of at least 95% intra-observer agreement. As for cases in which there was a history of malignancy, for such donors the main issue is the risk of cancer transmission. Several protocols have accordingly been developed to help stratify the probability of such an adverse event according to donors’ clinical, laboratory, and radiological findings.^12^^,^^31^ Thus, whenever the deemed risk of cancer transmission is too high to be overlooked, intraoperative histological examination of selected specimens becomes mandatory. Although the overall rates are low,^32^^,^^33^ cancer transmission to recipients is a recognized and documented phenomenon, often involving specific types of neoplasms.^34^ Not uncommonly, this may even occur with donors who have no suspicion of neoplasia at the time of donation according to screening protocols.^11^

Digital pathology enables pathologists to get urgent second opinions from experienced, remotely located pathology colleagues when dealing with difficult cases. Our validation set contained a wide spectrum of transplant cases of different specimens from varied organs that were suspicious for harboring malignancies. In summary, our validation study demonstrated non-inferiority of digital pathology using the NTP NED.Micro.DP® scanner to conventional LM for two different models of tablets for timely primary diagnostic use in transplantation pathology.

## Funding

Supported by a fund from Barone Rossi and Community of Albaredo d’Adige.

## Declaration of competing interest

Liron Pantanowitz serves as a consultant for NTP. The other authors have no relevant financial or non-financial interests to disclose.
