# Supplementary material for: Validation of portable tablets for transplant pathology diagnosis according to the College of American Pathologists Guidelines
Source: Acad Pathol. 2022 Jul 31;9(1):100047. doi: 10.1016/j.acpath.2022.100047 (PMC9356034; doi:10.1016/j.acpath.2022.100047)
Supplement: Multimedia component 1 [file mmc1.docx]

**Supplementary Table S1.** Hardware and software characteristics of the devices employed in the present study.

| **Device model** | **NTP NED.Micro.DP® microscope-based scanner** | **Microsoft Surface Pro X ® tablet** | **Samsung Galaxy Tab S7 FE 5G® tablet** |
| --- | --- | --- | --- |
| Screen size | 24.1” | 13" | 12.4" |
| Display | Eonis® (MDRC-2224 BL) LCD flat panel monitor | touchscreen brilliant PixelSense™ | LTPS TFT screen |
| Resolution | 1920x1200 | 2880x1920 | 2560x1600 |
| CPU | Intel® Atom™ x7-E3950 Quad Core @1.6 GHz (Burst 2.0GHz) | Microsoft SQ 2 ® software | Qualcomm Snapdragon 865 Plus software on an Octa Core processor |
| Memory | 8 GB RAM | 16 GB RAM | 4 GB RAM |
| Operating system | 64-bit Microsoft® Windows 10 Enterprise | Windows 10 Home on ARM | Android 10 system |
| Battery capacity | / | Up to 15 hours | Up to 14 hours |
| Networking | Gigabit Ethernet interface Intel® I210 or I211 Controller | Wi-Fi 5: 802.11ac | Wi-Fi 6 802.11 a/b/g/n/ac/ax |
| Bluetooth | / | 5.0 technology | 5.0 technology |
