# Supplementary material for: Validation of portable tablets for transplant pathology diagnosis according to the College of American Pathologists Guidelines
Source: Acad Pathol. 2022 Jul 31;9(1):100047. doi: 10.1016/j.acpath.2022.100047 (PMC9356034; doi:10.1016/j.acpath.2022.100047)
Supplement: Multimedia component 2 [file mmc2.docx]

**Supplementary Table S2.** Summary of cases sent for risk of malignancy assessment.

| **Case (N)** | **Sex** | **Age** | **Organ** | **Reference diagnosis** | **Risk category with LM** | **Risk category with WSI** |
| --- | --- | --- | --- | --- | --- | --- |
| 1 | M | 82 | Stomach | Nodule of 0.7 cm of mesenchymal spindle cell proliferation, with no necrosis, relevant atypia or atypical mitoses; GIST vs leiomyoma | negligible | negligible |
| 2 | M | 82 | Pancreas | Epithelial-lined cyst with no atypia | negligible | negligible |
| 3 | M | 77 | Prostate | Adenocarcinoma, Gleason 4+4 | acceptable | acceptable |
| 4 | M | 75 | Thyroid | Cystic benign nodule | standard | standard |
| 5 | F | 81 | Left kidney | Adipose nodule of 2.2 cm compatible with angiomyolipoma | standard | standard |
| 6 | M | 67 | Left lung | Fibrotic nodule of 0.8 cm under pleura; no evidence of malignancy | standard | standard |
| 7 | M | 89 | Prostata | Adenocarcinoma Gleason 3+4=7 | acceptable | acceptable |
| 8 | M | 63 | Thyroid | Follicular adenoma in multinodualr hyperplasia | standard | standard |
| 9 | M | 50 | Lymphnode | Lymph node with no evidence of malignancy | standard | standard |
| 10 | F | 82 | Gallbladder | Cholecystitis with lithiasis | standard | standard |
| 11 | M | 81 | Pancreas | Cyst lined by a columnar monostratified epithelium with papillary formations and mucous secretion, consistent with low grade IPMN | negligible | negligible |
| 12 | M | 87 | Jejunum | Intestinal diverticolar formation with no evidence of malignancy. | standard | standard |
| 13 | F | 64 | Duodenum | Pancreatic tissue ectopia | standard | standard |
| 14 | M | 75 | Lung | Bronchopneumonic focus with ascessualization | standard | standard |
| 15 | M | 75 | Right adrenal | Adrenal adenoma | standard | standard |
| 16 | M | 75 | Left adrenal | Adrenal hyperplasia | standard | standard |
| 17 | F | 62 | Lung | Hamartocohondroma | standard | standard |
| 18 | M | 65 | Thyroid | Micro- and macrofolicular thyroid in multinodular goiter | standard | standard |
| 19 | F | 50 | Uterus | Leiomyoma | standard | standard |
| 20 | M | 82 | Liver | Nodule consistent with liver hemangioma | standard | standard |
| 21 | F | 60 | Lung | Nodule of pneumocyte hyperplasia with chronic and acute inflammation with calcification; no evidence of malignancy | standard | standard |
| 22 | M | 79 | Lung | Fibrotic and calcified nodule with anthracosis; no evidence of malignancy | standard | standard |
| 23 | F | 73 | Pancreas | Serous cystoadenoma with low grade IPMN and low grade PanIN | negligible | negligible |
| 24 | F | 78 | Right ovary | Fibrothecoma | standard | standard |
| 25 | F | 78 | Liver | Poorly differentiated adenocarcinoma | unacceptable | unacceptable |
| 26 | F | 46 | Ovary | Serous cystoadenoma | standard | standard |
| 27 | F | 72 | Thyroid | Multinodular goiter; no evidence of malignancy | standard | standard |
| 28 | M | 49 | Bladder | Minimal chronic cystitis | standard | standard |
| 29 | F | 74 | Ovary | Serous cystadenomfibroma | standard | standard |
| 30 | F | 74 | Gallbladder | Chronic lithiasic cholecystitis with calcification | standard | standard |
| 31 | F | 78 | Ovary | Epithelioid neoplasia with high grade atypia and pleomorphism | unacceptable | unacceptable |
| 32 | F | 78 | Peritoneum | Infiltration of high-grade carcinoma | unacceptable | unacceptable |
| 33 | M | 88 | Thyroid | Multinoidular colloidal goiter | standard | standard |
| 34 | M | 88 | Bladder | Cystic cystitis | standard | standard |
| 35 | M | 65 | Prostate | Severe acute and chronic prostatitis | standard | standard |

GIST, gastrointestinal stromal tumor; IPMN, intraductal papillary mucinous neoplasm; LM, light microscopy; PanIN, pancreatic intraepithelial neoplasia; WSI, whole-slide imaging
