# Supplementary material for: Validation of portable tablets for transplant pathology diagnosis according to the College of American Pathologists Guidelines
Source: Acad Pathol. 2022 Jul 31;9(1):100047. doi: 10.1016/j.acpath.2022.100047 (PMC9356034; doi:10.1016/j.acpath.2022.100047)
Supplement: Multimedia component 3 [file mmc3.docx]

**Supplementary Table S3.** Summary of kidney biopsy cases.

| **Kidney** | **Sex** | **Age** | **Remuzzi score on LM** | **Remuzzi score on WSI** |
| --- | --- | --- | --- | --- |
| 1 | M | 82 | 6 | 6 |
| 2 | M | 75 | 4 | 4 |
| 3 | F | 81 | 7 | 7 |
| 4 | F | 82 | 4 | 4 |
| 5 | F | 74 | 4 | 4 |
| 6 | F | 74 | 6 | 6 |

LM, light microscopy; WSI, whole-slide imaging
