# Supplementary material for: Validation of portable tablets for transplant pathology diagnosis according to the College of American Pathologists Guidelines
Source: Acad Pathol. 2022 Jul 31;9(1):100047. doi: 10.1016/j.acpath.2022.100047 (PMC9356034; doi:10.1016/j.acpath.2022.100047)
Supplement: Multimedia component 4 [file mmc4.docx]

**Supplementary Table S4.** Summary of liver biopsy cases.

| **Liver** | **Sex** | **Age** | **Steatosis with LM** | **Score Ishak with LM** | **Score fibrosis with LM** | **Steatosis with WSI** | **Score Ishak with WSI** | | **Score fibrosis with WSI** | | **Discordance** |
| --- | --- | --- | --- | --- | --- | --- | --- | --- | --- | --- | --- |
| 1 | M | NA | 5% | 1 | 1 | 5% | 1 | 1 | | - | |
| 2 | M | 82 | 25-30% | 2 | 1 | 25-30% | 2 | 1 | | - | |
| 3 | F | 63 | 5% | 4 | 2 | 5% | 4 | 2 | | - | |
| 4 | M | 77 | 5-10% | 1 | 1 | 5-10% | 1 | 1 | | - | |
| 5 | F | 81 | 10-15% | 3 | 2 | 10-15% | 3 | 2 | | - | |
| 6 | F | 83 | 0% | 2 | 2 | 0% | 2 | 2 | | - | |
| 7 | M | 44 | 25-30% | 3 | 2 | 25-30% | 3 | 2 | | - | |
| 8 | M | 47 | 10% | 1 | 1 | 15% | 1 | 1 | | Minor discordance: the difference in scoring steatosis does not impact on allocation | |
| 9 | M | 86 | 5% | 1 | 1 | 5% | 1 | 1 | | - | |
| 10 | M | 75 | 5% | 1 | 1 | 5% | 1 | 0 | | Minor discordance: the difference in scoring fibrosis does not impact on allocation | |
| 11 | F | 81 | 5-10% | 3 | 2 | 5-10% | 3 | 2 | | - | |
| 12 | F | 55 | 0-5% | 2 | 1 | 0-5% | 2 | 1 | | - | |
| 13 | F | 66 | 5% | 2 | 1 | 5% | 2 | 1 | | - | |
| 14 | M | 68 | 0% | 2 | 2 | 0% | 2 | 2 | | - | |
| 15 | F | 78 | 10% | 1 | 1 | 10% | 1 | 1 | | - | |
| 16 | M | 89 | 5% | 2 | 1 | 5% | 2 | 1 | | - | |
| 17 | M | 63 | 15% | 1 | 1 | 10% | 1 | 1 | | Minor discordance: the difference in scoring steatosis does not impact on allocation | |
| 18 | M | 74 | 0% | 1 | 1 | 5% | 1 | 1 | | Minor discordance: the difference in scoring steatosis does not impact on allocation | |
| 19 | M | 70 | 10-15% | 2 | 1 | 5-10% | 2 | 1 | | Minor discordance: the difference in scoring steatosis does not impact on allocation | |
| 20 | M | 50 | 5% | 2 | 1 | 5% | 2 | 1 | | - | |
| 21 | F | 82 | 0-5% | 2 | 1 | 0-5% | 2 | 1 | | - | |
| 22 | F | 76 | 2% | 1 | 1 | 2% | 1 | 1 | | - | |
| 23 | M | 87 | 5% | 2 | 1 | 5% | 2 | 1 | | - | |
| 24 | M | 45 | 5-10% | 3 | 2 | 10% | 3 | 2 | | - | |
| 25 | F | 64 | 10% | 2 | 2 | 10% | 2 | 2 | | - | |
| 26 | F | 74 | 5% | 2 | 1 | 5% | 2 | 1 | | - | |
| 27 | M | 75 | 0% | 2 | 1 | 0% | 2 | 1 | | - | |
| 28 | F | 53 | 5-10% | 2 | 1 | 5-10% | 2 | 1 | | - | |
| 29 | M | 83 | 10-20% | 2 | 1 | 10-20% | 2 | 1 | | - | |
| 30 | M | 82 | 0-5% | 2 | 1 | 0-5% | 2 | 1 | | - | |
| 31 | M | 73 | 10% | 2 | 1 | 5-10% | 2 | 1 | | - | |
| 32 | F | 62 | 0% | 2 | 1 | 0% | 2 | 1 | | - | |
| 33 | M | 56 | 20-30% | 2 | 1 | 25-30% | 2 | 1 | | - | |
| 34 | M | 54 | 40-50% | 2 | 1 | 50% | 2 | 1 | | - | |
| 35 | M | 82 | 5-10% | 2 | 1 | 5-10% | 2 | 1 | | - | |
| 36 | M | 79 | 0% | 2 | 1 | 0% | 2 | 1 | | - | |
| 37 | F | 80 | 3-5% | 1 | 1 | 0-5% | 1 | 1 | | - | |
| 38 | F | 74 | 5-10% | 1 | 1 | 5-10% | 1 | 0 | | Minor discordance: the difference in scoring fibrosis does not impact on allocation | |
| 39 | M | 88 | 5-10% | 2 | 1 | 5-10% | 2 | 1 | | - | |

LM, light microscopy; NA, not available; WSI, whole-slide imaging
